# Green skills gap—A way ahead

**DOI:** 10.3389/fsoc.2025.1577037

**Published:** 2025-05-21

**Authors:** Terence Cook, David Elliott

**Affiliations:** Faculty of Science, Technology, Engineering and Mathematics, School of Engineering and Innovation, The Open University, Milton Keynes, United Kingdom

**Keywords:** energy, industrial decarbonization, green skills, just transition, sustainable employment

## Abstract

This study looks at what has been called the “skills gap” in the UK and the EU in key renewable energy and allied fields, factors that might slow the development of green energy technology. Within this emerging sector, new skills training is evidently going to be an urgent requirement. But, although there will be a need for new people coming into the industry to have the right skills, there will also be a need to upskill the current workforce. While the education system will play a role in increasing skills and raising awareness of green career paths, industry must also play a major role, for example via apprenticeship schemes and in-house training. Government can also help by providing support for training. Indeed, government strategic planning could include skill training requirements as a key factor in overall energy policy development. Moreover, this paper goes further and suggests that government should play a more strategic role, by formally requiring companies to provide the necessary training to be eligible for state funding of renewable projects. So, as well as looking at green skill gaps and their possible impacts, the paper also looks at the issues involved in trying to integrate green skills development into green energy expansion programmes, within the wider context of a “just transition”, the adaptive capacity of regions and communities to meet the challenges of a changing energy sector in terms of the need for “leveling up” via social and economic policies to reduce local and regional economic inequalities.

## 1 Introduction

In 2023,^1^ the global renewable energy sector witnessed a record increase in jobs, rising from 13.7 million in 2022 to 16.2 million. China led with an estimated 7.4 million renewable energy jobs, representing 46% of the global total. The EU followed with 1.8 million jobs, while Brazil had 1.56 million. The US and India each contributed nearly one million jobs. The strongest growth was seen in the solar photovoltaics sector, which accounted for 7.2 million jobs globally, with 4.6 million jobs located in China (IRENA/ILO, 2024).

However, there will also be jobs lost as the new green energy technologies replace fossil fuel-based technologies and the transition will not be a seamless one, with there being a range of impacts and some key issues being raised (Yang et al., 2024). Amongst them are likely green skills shortages, which potentially may slow progress. That issue is the focus of this paper, which we will be exploring initially in the UK context, and then more generally in the EU context, looking at what has been called the “skills gap” that is opening in new technology industries and the need to invest in the reskilling of workers to support regions in transition from carbon intensive industries.

Clearly, we must move beyond just looking at the social impacts of job losses as fossil fuel related employment falls, with modern socio-technical analysis also considering the adaptive capacity of regions and communities to meet the challenges of a changing energy sector. A shift in focus to sustainable employment is now perceived as vital for the “Just Transitions” project to be implemented, with links also being made to “leveling up” social and economic policies to reduce historical and geographical inequalities.

## 2 Green jobs and green skills

So far, over 270,000 jobs have been created in the UK in its transition to green energy technology (ONS, 2022), and the Government is aiming to deliver on its flagship goal of creating two million green jobs by 2030. Similar targets exist for the EU. However, it is not clear exactly what is meant by “green jobs”. In principle it could mean any job that has a positive environmental element, which, for example, might include a range of nature conservation and rehabilitation jobs. The International Labor Organization also offers a quite broad definition. It says green jobs “*produce goods or provide services that benefit the environment, for example green buildings or clean transportation”* (ILO, 2016). A somewhat more limited definition would be to reserve the term for jobs that involve reducing carbon emissions. On that basis it becomes a little easier to see what is meant by green jobs—jobs in green energy supply and energy conservation.

The definitional issue becomes a little more tortuous when we look at the skill requirements for green jobs, the focus of this paper. A briefing note in 2024 from POST, the UK Parliamentary Office of Science and Technology, says that green skills can be defined as “*the knowledge, abilities, values and attitudes needed to live in, develop and support a society which reduces the impact of human activity on the environment”* (POST, 2024). That, it admits, is very broad, well beyond a simple focus on technical skills for jobs that play a major role in reaching net zero greenhouse gas emissions by 2050. But green skills can involve more than just engineering know-how: as Prof. Stephen Peake and Dr. Victoria Hand from the Open University have suggested, they may also involve, amongst other things “*ecological literacy and nature centric design, systems rethinking and redesign for integrated services, circular and closed loop resource cycling”*. They add that, given climate risk multipliers from extreme impacts such as heatwaves, droughts, wildfires, and flooding, “*every job has the potential to be a green job”*, for example in disaster management, prevention, risk reduction, mitigation, or nature restoration, as well as community resilience and vulnerable community care (Peak and Hand, 2023).

That may well be the case, in terms of what is needed in relation to general educational provisions, but Peake and Hand also offer are more limited definition: the skills needed for “*resource efficiency and climate resistant redesign of machines, cities and infrastructure”*, with the emphasis on energy technology, including renewable energy systems.

In fact, most of the studies and reports cited in this paper focus primarily on the later aspect, with, for example, an article in the Engineer talking of “*high-level electrical, technical, and engineering skills”* in relation to renewable energy supply technology, but with this demand being “*further amplified by the need to upgrade the power network and establish battery storage sites”*. It also says that there will “*significant demand for skills in advanced digital specializations, such as data analytics, artificial intelligence, robotics, software development, and machine learning”* (Maxted, 2025).

The methodology used by the authors of this paper includes a review of existing literature, field research of UK and European renewable energy projects, inclusion of primary data from a 2024 survey of energy and utilities sector green jobs and skills provided by permission of Economist Insight, and a focus on defining green skills rather than relying on the limitations of existing job classifications data. The measurement of the employment effects of renewable energy is complicated. Surveys that have been undertaken to measure the direct employment effects of job creation from renewables are calculated relatively easily, but the measurement of indirect or induced employment effects is more difficult, as the causality is not explicit. It is difficult to capture indirect or induced employment through surveys alone, not least when the time gap between a project and new job development is taken into consideration, and because the renewable technology sector changes so quickly.

Assessing employment impacts of renewable energy development presents several methodological challenges which need to be addressed. There are clear limitations of what can be measured. Econometric studies with input-output calculations often rely on labor market data such as SIC (Standard Industrial Classification) codes to collect statistical data and to analyze trends across different industries and provide structural insights into the UK economy. Adopting a skills-based approach allows impacts to be captured and demonstrated in real time that is more representative of a new generation of a green skilled labor force. Agreeing on the methodologies for measurement and gaining an understanding of indirect and induced impacts is a priority.

A focus on skills rather than occupations when considering the net zero economy reflects the relatively new types of activity that will continue to evolve as new technologies become available. Emerging industries do not conform to the traditional industry classifications, so relying on Standard Industrial Classification (SIC) codes to identify a firm's primary business activity can be outdated when searching for innovative new green businesses. The SIC has been slow to recognize new and emerging industries, such as software and AI. Green skills and the net zero economy involve new types of technology and new industries based on research and development so do not conform to traditional industry classifications. Focusing on “green skills” rather than specific “green occupations ” provides an understanding of how the evolving needs of the green economy is adapting and developing.

Some might include nuclear skills in the green skill definition. However, there are only a few specifically *nuclear* skills, and they are in short supply—most of the workforce involved with nuclear projects have more general skills that can be used for any area of energy engineering.

**Figure 1:**
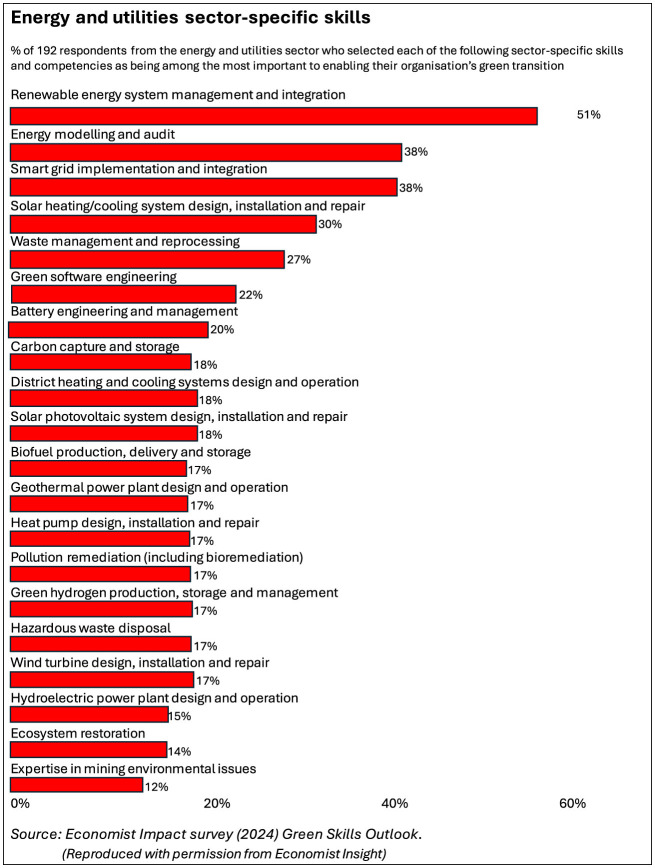
Energy and utilities sector-specific skills. Reproduced by permission from Economist Insight.

It is also likely that, in any case, nuclear will remain a relatively small contributor to global energy supply—in most global scenarios renewables dominate. So nuclear skills as such will not be covered in this paper. Neither will the broader managerial skills that, “*in addition to technical roles”*, are mentioned in the article in the Engineer, such as “*expertise in project management, legal and policy advisory, environmental impact assessments (EIA), stakeholder management, land management, and bidding”*. Instead our focus is on the more limited range of engineering and design skills associated with renewable energy supply and use and power system management. There is certainly plenty to do in that area of the green economy, including in terms of the energy used in housing and transport. See Box 1 for a summary of the green energy technologies as covered in this paper and some data on the breakdown of associated skills and an estimate of prospective green job expansion. Figure 1 shows the results of an **Economist Insight** survey of energy and utilities sector green jobs and skills, which also includes some environmental restoration, pollution clean-up and waste disposal jobs not green energy related. But even with those included, clearly, renewables dominate (Economist Impact, 2024).

Box 1 **Green energy technologies.**

**Renewable energy sources—**wind, wave, tidal, biomass, solar, geothermal, hydroelectric, mostly used for electric power generation, but solar and biomass can be used for heat supply and green power, green gas and green hydrogen (see below) can be used for transport

**Ancillary activities for green energy systems—**power transmission and energy storage

**End use efficiency—**energy waste reduction via insulation, heat pumps, low energy systems

**Hydrogen gas generation** by electrolysis using renewable power (green hydrogen).

The following are not seen as green energy sources:

**Blue hydrogen** production using CO2 from fossil fuel combustion with Carbon Capture and Storage to make it lower carbon, also **power** direct from combustion of fossil fuel with CCS

Low carbon power (and heat and hydrogen) made using **nuclear** sources.

## 3 Green skills gap in the UK

The UK Climate Change Committee (CCC) says that the net-zero transition will transform many parts of the UK economy and provide an opportunity for growth in high-quality jobs and distribute economic opportunities across the UK. But it warns that job growth is not guaranteed, and upskilling will need to be a key political focus. The then UK Green Party MP Caroline Lucas said that “*Ministers can't simply wave a magic wand and expect green jobs to appear or rely on the market alone to dream up a solution. We need to see political will across Whitehall, to properly invest in reskilling and retraining workers in the green industries of the future”*.

There certainly do seem to be problems. In a UK Institution of Engineering and Technology survey in 2020, 88% of engineering employers with a sustainability strategy said that their business needed new skills to deliver, while 48% of engineering employers reported that “*applicants lacking the necessary technical skills is a difficulty they face when recruiting”*, with 68% going onto say it's “*specialist skills/knowledge needed for the role that's most lacking”* (IET, 2020). Similarly, in a workforce survey of 1,000 UK energy professionals in 2023, one-third of the respondents believed they had the skills needed to adapt to any future changes in the energy industry, while 26% said they didn't know how to access training that will allow them to adapt their existing skillsets (City & Guilds/Engineering UK, 2023).

A report in 2024 by the Royal Academy of Engineering and National Engineering Policy Center says that “*about an additional 200,000 workers are needed by 2030 to meet expansion demand on top of those required to replace the existing aging workforce”*. However, instead, it noted that there were “*declines in key parts of the workforce and high rates of hard-to-fill vacancies in critical occupations such as engineering project managers, electrical & mechanical engineers, engineering technicians, welding and engineering construction trades (e.g., crane drivers, steel erectors) alongside a wide range of other occupations key to energy transition”*. At the same time, it said “*we are seeing stagnation or reduction in the supply of young people into these roles. Apprenticeships in engineering and manufacturing have seen a 34% fall over the last decade. Engineering degrees by UK students have remained fairly static at about 28,000 per year over the same period, and while some disciplines such as mechanical engineering have seen growth, electronic and electrical engineering have seen a decline”* (RAE/NEPC, 2024).

So, there does seem to be a skills gap opening in the UK, with an Engineering Construction Industry Training Board's Sectoral Workforce Census report noting that electrical and mechanical fitters, pipefitters, platers, non-destructive testing technicians and planners were among the roles that are proving most difficult to recruit (ECITB, 2025), and a review of offshore wind power employment noting that, “*as well as competing with each other for talent, the renewables industry is competing with other sectors for the same highly sought-after skill”*. It concluded that change was clearly called for: “*Without an upskilled workforce, there will be no energy transition. The increasing ambitions mean that demand for workers is rising faster in the renewable sector than in other industries, giving the energy sector both an incredible opportunity and a growing challenge”* (Derrick, 2024).

## 4 Change options—and barriers

The 2024 POST review says that, in the UK “*sectors such as power generation, construction, waste and resources are likely to see growth”* and need “*a significant update in skills as part of the net zero transition”*. In terms of what needs to be done, the UK government's Green Jobs Taskforce, Final Report 2021, said that employment in the UK's transition to net zero should be enhanced by (1) Business commitment to offer training to the workforce, (2) Improving the Apprenticeship Levy that works for all businesses of all sizes, (3) Employers linking to education providers to develop short courses for industry.

Although that implies action in a range of areas, POST noted that, since it was still a newly emerging field, for the moment, the transition “*will mainly involve upskilling existing workers”*. To that end, it says that “*Further Education (FE) colleges, formal training and qualification providers, employers, and Higher Education providers will play key roles in delivering this training”* (POST, 2024).

A 2024 Imperial College *Futures Lab* briefing paper looks in some detail at barriers and opportunities for expanding low-carbon job competencies (McCluskey et al., 2024). Using three sectoral case studies, the paper investigates challenges and opportunities for improving skills and training. Firstly, it shows how the *building energy retrofit* sector faces a significant shortage of skilled workers, particularly in heat pump installation, energy efficiency measures, retrofit coordination, and digital roles. Despite the potential to create 120,000–230,000 new jobs by 2030, it says “*inconsistent policies and funding have hindered private investment in training”*. Secondly, the *offshore wind* sector is forecast to employ over 100,000 workers in 2030, compared to 32,000 in 2022. But it says “*offshore wind struggles with skills gaps in electrical, digital, consenting, and marine roles, relying on experienced workers and those from other industries to fill these gaps”*. Thirdly, the paper claims the *electric vehicles* sector “*could generate at least 80,000 new jobs over the next 10-15 years”* but says that this “*is contingent on gigafactory development, with key skills needed in charging point installation, vehicle recycling, battery manufacturing, and electrification engineering”*.

Most of these cases involve expanding training for specific green energy technologies and electrification, with digital technologies helping out, but the report says that “*not all industrial decarbonization can be achieved through direct electrification, and particularly across hard-to-abate industries, decarbonization will depend on the development of hydrogen and CCUS sectors”*.

It notes that “*growth of these sectors is considered highly conditional, subject to the competitiveness of international markets, the availability of skilled labor, and levels of investment,”* but the CCC estimates that “*these industries could create between 1,500 and 97,000 new jobs by 2030”*. It adds that “*the current offshore oil and gas workforce is expected to provide a large number of skills required in these sectors”*.

That is good news, although there are debates over whether Carbon Capture and Storage is viable at scale, and on whether the focus should be on CCUS, producing low-carbon so called blue hydrogen using CO_2_ from fossil fuels with CCS, or on carbon-free green hydrogen, produced using renewables to power electrolysers. Either way, making it happen will not be easy. It is interesting in this context that there has recently been a call for £1.9 bn a year to help oil and gas workers move into clean energy, with the Green Jobs Taskforce also estimating that “*the low-carbon transport sector could create 78,000 new jobs by 2040, including 24,500 in battery manufacturing, 43,500 in the battery supply chain, and 10,000 in EV manufacturing”* (EDP, 2024). That may be optimistic, especially given more recent UK experience, for example with the failed Britvolt battery plant in Northumbria (Denten and Manning, 2024), and certainly there can be problems for projects like this, as we will see later in the case of Northvolt, in Sweden.

Looking to the way ahead, the Futures Lab identify a series of specific barriers facing the ongoing job transition, including “skills transferability” barriers between sectors, and “mobility” barriers. For example, workers transferring from oil and gas into offshore renewable roles and their respective locations, that latter linking up to regional barriers and issues. It says “*UK regions with a higher concentration of energy-intensive industries, such as the North East, Yorkshire & the Humber, and the West Midlands, stand a higher chance of being negatively affected by the transition”*, since “*they are often also those whose economies have seen the least growth in recent decades”* and “*are also likely to have less capacity and resources to be able to provide adequate re-skilling support.”*

And finally, it looks at diversity barriers. It says “*the current energy sector is predominantly represented by white male workers…Unless active measures are taken to support underrepresented groups joining the Net-Zero energy workforce, occupational gender & ethnicity gaps are likely to persist”*.

Overall, to deal with issues like this, it calls for building “*closer links between high- and low-carbon energy sectors to create direct routes into new jobs”* and improving the financial arrangements for training and apprenticeships. And more generally, it says we should “*introduce a national Net-Zero Skills Commission to take on monitoring, research and advisory roles to support development of skills for the Net-Zero transition in England”* (Futures Lab, 2024).

## 5 Some proposals for action

There have also been some specific proposals for way to move things along. For example, the Labor government has set up an initiative, “Skills England”, which aims to develop a nationwide skills strategy and provide levy funds for businesses to spend on training and “future-proofing” the workforce, with green skills being key, especially for younger people now entering the jobs market (Mittal, 2024). Research has found that only 5% of the ‘Gen Zs' in the workforce believe they currently have the green skills required to help drive a net-zero transition, but nearly 78% of them believed that if they were offered training, they would improve key green skills that would assist with corporate efforts to decarbonize (Mace, 2024).

There have also been proposals for energy skill passports, to enable cross-sector recognition of energy industry expertise and training (Green, 2024), in particular designed to help enable the retraining and reskilling of oil and gas workers for clean energy roles (Keating, 2024). A preliminary test version has created career pathway information for over thirty oil and gas roles and entry routes into the wind industry. Many companies are also setting up support schemes. For example, E.ON's Chief People Officer, Chris Norby, told the 2023 Westminster Employment Forum conference that E.ON had hired 4,000 people over the past 3 years, supported with a skills learning infrastructure, with 180 re-skilling apprenticeships including digital technology capabilities (WEF, 2023).

Of course, it could be argued that, to an extent, market forces will sort out some of this, the labor market responding to demand for people with the necessary green skills. For example, renewables firms are increasing salaries due to green skills shortage (George, 2024). The latest Global Energy Talent Index, including the results of research among 12,000 energy professionals across 149 countries, suggest that the growing green skills gap is making workers with renewable energy expertise ever more sought after. 51% of low-carbon energy workers received a pay rise in 2023, with 24% of the workforce saying its rise was above 5%.

Then again, industry could play a more active role, apart from just from adjusting pay rates. A recent survey of what industrial leaders think about the energy transition by “Economist Impact” found that 79% of the 1000 surveyed anticipated more opportunities than challenges, but believed that responsibility for driving the transition rests with the private sector rather than policymakers. Some are certainly trying to make changes. For example, the survey found 55% of the companies in its survey were actively reskilling and/or upskilling their existing workforce, and 51% were targeting the recruitment of new employees who possess relevant green skills (Economist Impact, 2024).

However, it could also be argued that governments could play a more strategic role in terms of green skill development within energy and climate policy formulation. For example, it has been suggested that countries should include plans for building a climate-ready workforce in their Nationally Determined Contributions (NDCs) for energy under the Paris Climate Agreement (LinkedIn, 2024). Support for skill training could also be more directly stimulated by governments. For example, they could formally require companies to provide the necessary training to be eligible for state funding of renewable projects.

There have been relevant initiatives along these lines emerging in the EU. For example, the EU “Just Transition Fund” invests in the reskilling of workers to support regions in transition from carbon intensive industries, with eligibility requirements as a condition for investment. It's aided by the European Investment Bank (EIB), with JASPERS, the EC/EIB funded “Joint Assistance to Support Projects in European Regions” programme, playing a key role.

The recent adjustments of the EU State Aid system may also provide an opportunity for skills-related issues to be taken account of in new green energy subsidy arrangements (EC, 2024a), while government strategic planning could also include skills training as a key factor in any energy funding taxonomy: a green skills taxonomy could contribute to a common understanding of which skills are needed for a successful and fair green transition.

Skill upgrades are clearly seen as urgent in the EU and not just in terms of energy jobs. For example, the recent EC Strategic Foresight report has “Demand for Future Skills” as the priority, saying that growing skills disparity could impede the twin green and digital transitions (EC, 2023).

There is clearly much going on in the EU, the UK & elsewhere in this general area, as shown in a new IPPI overview exploring the links between green jobs & the Just Transition, and advocating for “*a holistic approach that not only accelerates the transition to a green economy but also ensures that this transition is inclusive, just, and beneficial for all”*. But it warns that “*no one size fits all—there are diverse paths to green job creation”* (IPPI, 2024).

Even so, it urges policy makers to be bold and back green job creation, since it says the associated policy risks are “relatively low”, and the potential outcomes are very attractive: such as “*a transition to a greener economy that not only addresses the challenges of climate change and environmental degradation, but also cultivates inclusive, prosperous societies with aligned socio-economic objectives”*. That sounds a worthwhile goal—but not an easy one to achieve fast, given the historical and geographical imbalances in resources and power.

## 6 Leveling up

While the energy transition may lead to worsening social and economic imbalances, it should be possible to avoid this and indeed to use the transition to “level up”, by sector and locational planning, and creating new green jobs in key areas. In this section we will look at examples of what has been called “just transitions”, in which support has been given to vulnerable groups and regions in the EU which are dependent on carbon intensive industries, to help them to make the green job transition.

The most obvious problem areas are those involving coal mining, coal-fired power station operation, oil processing, steel-making and other heavy industry. These are examples of negative externalities of green transition policies that impact European regions with high carbon intensive industrial sectors. Certainly, significant job losses will occur in coal mining regions in the EU, such as Upper Silesia, the heart of Poland's mining industry and South-West Otenia in Romania. In Upper Silesia as many as 41,000 job losses are expected.

There are some examples of good responses to this type of challenge, for example in Katowice in Poland, once a major center for coal mining and heavy industry, but now, in a post-industrial revamp, something of a show case for green energy and local regeneration.

It hosted the COP 24 United Nations Climate summit. Crucially it has an Engineering Training Center for advanced reskilling that has trained 70,000 people. Along with a new cultural center and museum, it is claimed to have shifted from being “*an industrial and mining city into a business and cultural city”* (Moloc, 2018).

In another showcase example, new green industries including green steel production and green hydrogen have started up in the small town of Boden in Northern Sweden. Strega is replacing coal with green hydrogen and building the first large-scale commercial green steel plant in Europe, electrifying the steel-making process using power from a giga-scale electrolyser powered by renewable electricity from a hydro plant on the Lule River.

Strega says it can “*tap into a strong local and regional talent pool and combine it with international expertise and next-generation technology”* (Strega, 2024).

As well as backing projects like this, the EU has made a more general response to local and regional employment problems, via the EU Cohesion Policy (EC, 2024b). That seeks to address the geographical, economic, and social inequalities that exist across Europe, with the “Just Transition” platform policy providing the basis for addressing the negative effects on social cohesion from regional location impacts on employment and supply chains because of the green transition in economically lagging regions across Europe. Where inequalities are created in already vulnerable regions there is certainly a threat to cohesion across Europe and the risk that the green transition will lead to a backlash against “Net Zero” policies.

The green transition is a shared challenge. Training and education programmes need to be clearly synchronized with government and industry since investment in skills provision is critical to ensure the skill to design and install the new technologies. It is a critical component for maximizing employment growth to meet the scale of demand. The government framework should be multi-level enabling the role of local and regional authorities to lead the transition.

The connections between technical and vocational education, apprenticeships, courses for technicians and skilled craft workers with career pathways in industry is needed to develop the specialist skills and knowledge associated with low-carbon technologies. Monitoring the flow of employment is needed as new skills for innovative processes and new technology industries change the employment landscape. The link between government policy makers, education and training services and employers needs to be responsive to what facilitates new employment in these sectors to ensure that investment in new technologies is able to realize the greatest employment effects.

Economic diversification and reskilling workers clearly can be helpful to counteract the negative local economic impact of the green transition by creating new opportunities, new jobs, and new skills, with new clean and resource efficient technologies being a key focus. For example, in the transport sector, it is argued that new projects in advanced battery technologies can contribute to reducing the social, employment, economic and environmental impacts of the transition in lagging regions.

As a case in point, the Swedish battery maker Northvolt attracted billions of euros investment from the EU and orders from major car manufacturers Volkswagen, BMW, and Volvo to set up a lithium-ion battery Gigafactory, in Sweden's rural north in the small town of Skellefteå, with 72,000 inhabitants 200 km south of the arctic circle. It was seen as a flagship project for Sweden's green industrial revolution, and as Europe's first home-grown battery Gigafactory (EIB, 2018). However, there was a lack of skilled labor for such an enterprise, and it also has had financial problems. With the global market for batteries (led by China) becoming increasingly competitive, Northvolt had to scale back its plans (Northvolt, 2024).

Clearly, developing local initiatives in new areas of technology is not always easy and progress will be constrained if the local skills supply is limited, as it often is in isolated areas. But conversely that means that support for training (and new jobs) can change things and attract young people to stay—they may no longer feel pushed to leave the region.

However, improving the local supply of skilled and well-educated workers may still not achieve growth if those workers then chose to migrate to more prosperous and economically dynamic regions. The brain-drain (emigration of qualified people), and depopulation of areas that Europe has been suffering from, is responsible for new and growing disparities as various regions populations age and fall behind in terms of population size and skills.

Several EU countries are expected to see their populations decline by more than 15% by 2050, including Bulgaria, Croatia, Hungary, Latvia, Lithuania, and Romania (Europa, 2024a).

This seems to be the case in Sisak Moslavina county in Central Croatia. Sisak-Moslavina is leveling up with the EU Joint Transition Fund. It is a region formerly dependent on metallurgy, oil-refining and the production of chemicals and a tradition of heavy industry going back to the Soviet era, employing thousands of workers. But now, with the help of support under the EU Cohesion policy, it is changing with new high-tech investment and an ambition to become the “Silicon Valley of the Balkans”—for example it is turning into a hub for the European videogaming industry, preventing brain-drain, and attracting young people. To move thing along there is the Regional Skills Center in Sisak, a “knowledge center” in the fields of electrical engineering and computing. The project, worth €10 m and co-financed by EU funds, aims to make the people it trains more competitive on the labor market (Europa, 2024b), and, more specifically on the green tech side, another EU backed local project aims to help local businesses transition to a green economy (Startup Europe, 2023). Delegates from EU Member States discussed these issues at the 10th Just Transition Platform Conference organised by the European Commission and hosted by Commissioner Elisa Ferreira DG REGIO 15–17 October, 2024 (EC, 2024c).

Developing an ecosystem where innovation, education and training can deliver quality jobs is a widely shared requirement throughout Europe and, in response, EU's “Just Transition” fund is being used to address labor and skills shortages in strategic sectors and enhance the long-term competitiveness of regions (EC, 2024d). In parallel, the Strategic Technologies for Europe Platform (STEP) has been set up by the EU to support European industry and increase investment in technologies for the green transition. STEP supports projects growing the skills necessary to develop critical green technologies. It recognizes regional weaknesses, with there often being insufficient skilled staff to carry through research and development. To help, the programme supports universities to research and implement new technologies, creating value chains by linking up with, industry, and research training institutes. For example, it is trying to identify breakthrough technologies that could accelerate the energy transition, such as in the health care sector, a major energy intensive sector (EC, 2024e).

Although initiatives like this can involve a sectoral approach, as we have seen, there is also often a local “spatial” component to green job and green skill development, with leveling up often involving local or regional initiatives, along with locational planning system changes. In that context, it is interesting that, in the UK, where there are strong pressures for leveling up (Martin et al., 2022), in addition to pushing ahead with sector focused initiatives like the skills passport system (Beament, 2024), and the creation of an Office for Clean Energy Jobs, the new UK Labor government has “work in progress” on a new strategic and spatial planning framework (ECITB, 2025). It has also tasked the newly established National Energy System Operator with creating a strategic spatial plan that maps where net-zero infrastructure such as renewables generation and storage should be located across the UK.

Clearly, the transition is about more than just transforming energy intensive industries with renewables. It is much wider, involving not just green jobs and skill creation, but also planning to ensure the effective distribution of green technology/industry clusters and the creations of regional economic and employment plans, with local community benefits in mind. As we have seen in the examples from around the EU above, new green energy projects certainly can have positive local impacts on local communities, and there are also many examples of community benefits from green power initiatives in the UK (Energy UK, 2024).

In summary then, when looking at transition options, a range social issues therefore must be explored, as well as just technical issues, with relevance across all sectors of the economy, as well as the wider community. For example, energy poverty has increased. In 2022, over 41 million people in Europe were unable to keep their homes adequately warm (EC, 2023). So, a key criterion for any new energy technology is that it will reduce the cost burden, with energy saving skills being especially important (European Parliament Briefing, 2023). In addition, a “place-based” approach to labor market transitions needs to be adopted where-ever possible (Weller et al., 2024), so that, for example, people can live and work in the regions they come from. That may be hard in regions suffering decline. But, with leveling up in mind, as the Futures Lab study argued, efforts need to be made to make the transition as socially fair as possible. For example, it said that there is “*a case for targeted funding for SMEs who cannot afford to send staff to be trained or take on apprentices”* (Futures Lab, 2024). At the same time, we also have to accept that employment patterns are changing, requiring flexible reactions and nimble responses from all the players, including educationalist, politicians, employers, unions and workers. It may be challenging to ensure that training needs are met, but even more so that stable new green careers and employment patterns emerge.

## 7 Conclusion: green energy strategy

Renewable energy is creating new jobs, replacing those lost as fossil-fuel related employment falls. However, as we have seen, it is not a seamless transition, and it may take time. And although some of the skills involved with building and operating renewable energy systems are like those that some people already have, not all of them are the same. Some of the new green energy technologies need people with specialist skills and, as we have seen, some of these may be in short supply.

As we have seen, that is already an issue in the UK and EU and it is also an issue globally, with a report from LinkedIn warning that global demand for green talent increased by 11.6% between 2023 and 2024, compared to just a 5.6% rise in available talent (LinkedIn, 2024).

This study has reviewed some of the issues and some of the recommended solutions, including approaches that governments might make at the strategic policy level. For example, it was suggested that governments might formally require companies to provide the necessary training to be eligible for state funding of green energy projects. Indeed, government strategic planning could include skills training as a key factor in any energy funding taxonomy.

The European Union has developed a taxonomy/classification system to guide energy investment decisions, identifying which types of technology it thought should be included. Clearly renewable energy was central to the EU plan, but, after much debate and conflict, nuclear was included, along also with gas as in interim option. The debate continues, and that has some relevance in terms of the skills focus of this paper. For example, although, as noted earlier, the nuclear share of total global energy supply is likely to remain small, it could be argued that, in terms of a “skills taxonomy”, it would nevertheless still be a waste of talent and skill to focus on nuclear projects: any available skills could arguably be more productively be used elsewhere, on renewables and energy system upgrade work (Präger et al., 2024).

Some say technology choice issues like this should be left to the market, although, at least on current showing, in most places, that would lead nuclear to be rejected as (amongst other things) being too expensive, in preference to renewables, the costs of which are falling (Creutzig et al., 2023). There may however be other strategic factors to consider which would have relevance in terms of any potential skills taxonomy. For example, the employment impact of energy investment. That may follow the economic cost, since expensive technologies tend to create more jobs, all other things being equal, although not all other things are equal: some types of renewable energy technology can be very labor intensive, biomass production for example. Energy conservation jobs similarly. They can also be created quickly. By contrast, nuclear is very capital intensive and slow to develop. But should we develop a taxonomy based on optimal job creation? It would be contentious to develop and impose criteria like this, even more so to use skill development issues as the main determinant. Instead, in this paper, we have proposed a less dirigiste approach, introducing a skill training requirement in investment funding decisions. That may not escape the need to assess the social, economic, and environmental implications of energy decisions, but it might add a more pragmatic element, enabling a more rapid transition.

Nevertheless, it will not be easy. Moreover, it will not be sufficient just to provide skills and training, we also have to try to ensure that there are jobs to go to- an even tougher political and economic requirement. A sustainable and just future requires more fundamental reshaping of economies and goes beyond the limits of existing vocational education, training programmes, and new hiring routes. Companies can help, but ultimately it falls to government to develop policies that try to create new jobs in agreed new areas and sectors, and that can be difficult, given political differences, as we are seeing in the USA at present.

Certainly, we must recognize that the transition will be difficult in some cases, for example in areas already suffering from depravation, as we saw earlier, and as has also been found in the USA (Lim et al., 2023). It can be hard to phase out an old but well-established industry. But it may be possible to make a technological shift to uplift the region, with new companies attracted to the area, leading to a deep transformation of its economy in a policy led transition. Though there may then be a need for accompanying policies that strengthen the social dimension, change models that ensure that growth is socially inclusive, and that create a positive outlook by creating new jobs, and more money for upskilling and reskilling. There will also be a need for better prediction of the skills needed to enable people to move to new opportunities via retraining if needed. There may also be a need for increased welfare, like affordable housing in impacted areas to alleviate social and economic costs. All in all, the social side is likely to be even more challenging than the technical side. But both will be needed for a full, fair, and effective transition.

However, looking on the positive side, the transition is good news for those who already have green skills. As the 2024 LinkedIn Green Skills report notes, jobseekers with green skills are seeing a 54.6% higher hiring rate than the workforce average globally. Hopefully, the renewables boom will continue around the world, so the need for enhanced green job supply and skills training will be even greater, allowing even more people to share in the benefits of responding positively to the threats of climate change and eco-system damage.

## Footnotes

^1^Parts of this paper are based on coverage of the issues in the *Renew* newsletter: https://renewnatta.wordpress.com/.
